# Folding-Based Electrochemical Aptasensor for the Determination of β-Lactoglobulin on Poly-L-Lysine Modified Graphite Electrodes

**DOI:** 10.3390/s20082349

**Published:** 2020-04-20

**Authors:** Olaya Amor-Gutiérrez, Giulia Selvolini, M. Teresa Fernández-Abedul, Alfredo de la Escosura-Muñiz, Giovanna Marrazza

**Affiliations:** 1 Department of Chemistry “Ugo Schiff”, University of Florence, Via della Lastruccia 3, 50019 Sesto Fiorentino (FI), Italy; amorolaya@uniovi.es (O.A.-G.); giulia.selvolini@unifi.it (G.S.); 2 NanoBioAnalysis Group, Department of Physical and Analytical Chemistry, University of Oviedo, Julián Clavería 8, 33006 Oviedo, Spain; alfredo.escosura@uniovi.es; 3 BioNanoAnalytical Spectrometry and Electrochemistry Group, Department of Physical and Analytical Chemistry, University of Oviedo, Julián Clavería 8, 33006 Oviedo, Spain; mtfernandeza@uniovi.es

**Keywords:** β-lactoglobulin, folding-based aptasensor, poly-L-lysine, conducting polymers, methylene blue

## Abstract

Nowadays, food allergy is a very important health issue, causing adverse reactions of the immune system when exposed to different allergens present in food. Because of this, the development of point-of-use devices using miniaturized, user-friendly, and low-cost instrumentation has become of outstanding importance. According to this, electrochemical aptasensors have been demonstrated as useful tools to quantify a broad variety of targets. In this work, we develop a simple methodology for the determination of β-lactoglobulin (β-LG) in food samples using a folding-based electrochemical aptasensor built on poly-L-lysine modified graphite screen-printed electrodes (GSPEs) and an anti-β-lactoglobulin aptamer tagged with methylene blue (MB). This aptamer changes its conformation when the sample contains β-LG, and due to this, the spacing between MB and the electrode surface (and therefore the electron transfer efficiency) also changes. The response of this biosensor was linear for concentrations of β-LG within the range 0.1–10 ng·mL^−1^, with a limit of detection of 0.09 ng·mL^−1^. The biosensor was satisfactorily employed for the determination of spiked β-LG in real food samples.

## 1. Introduction

Nowadays, allergy to food is a very important global health problem, thus an emerging topic when talking about food safety [1,2]. Allergy to food has been recently defined by the European Academy of Allergology and Clinical Immunology [3] and it involves an immune reaction deriving from the intake or other type of interaction with specific food [4,5]. Food allergy is a type I hypersensitivity immunological response against ingested substances that can result in serious health problems [6,7]. The reactions can be caused when even little quantities of food are consumed, usually between 10 and 100 mg [4,8]. It is known that this kind of hypersensitivity is having an impact especially in developed countries: between 1% and 3% of adults and between 3% and 8% of children are affected all over the world [9,10]. Symptoms vary from person to person, but can involve, for example, digestive disorders, circulatory or respiratory symptoms or skin irritations, among others. Moreover, an allergic reaction can also provoke in some cases anaphylactic shocks, that are life-threatening situations [11].

Allergens are mainly divided into eight different groups: fish, shellfish, wheat, milk, eggs, soy tree nuts, and nuts [12]. A study carried out in 2014 by Nwaru and collaborators about the incidence of the most popular food allergies throughout Europe [13] revealed that allergy to cow’s milk is, nowadays, the most frequent food allergy, especially in early-age children, implying a percentage of around 6% in the youngest population [14,15]. Immunoglobulin E (IgE) normally mediates cow’s milk allergy, and the symptoms usually occur within 2 h after milk intake [16]. In whey from cow’s milk, one of the most important proteins considered to be an allergen is β-lactoglobulin (β-LG), which is classified within the lipocalins, a group of binding proteins with small ligands [17]. Its quaternary structure, identified in 1997 using X-Ray diffraction [18], is dependent on the pH. For example, at pH values between 5.2 and 7.0, it is a dimer, and its molecular weight (MW) is around 37 kDa. However, at pH values over 8.0, it exists as a monomer, and its MW is approximately 18 kDa [19]. This protein has a great potential as milk allergen because it is remarkably stable and it is not present in human milk [20], and, apart from that, it is regularly used as an additive in lots of food products [21].

The development of analytical approaches used to detect and quantify food allergens has become increasingly significant [22] because labeling in food is crucial for the consumers, in order to prevent major health problems. The analysis of allergens in food samples [23] is usually carried out by immunoassay-based approaches, such as enzyme-linked immunosorbent assays (ELISAs) [11,14,24] or lateral-flow immunoassays (LFIAs) [25,26], as well as DNA-based methods, which detect the genes encoding for the proteins using amplifying methods, for instance, the polymerase chain reaction (PCR) [27,28]. Proteomic tests have been also used for the determination of allergens [29]. Although these methods are very sensitive, they are time-consuming and the instrumentation needed is very expensive. Because of this, there is an increasing interest in developing new analytical tools which are faster, simpler and with a lower-cost, also keeping good analytical characteristics [3,30]. Hence, electrochemical biosensors satisfy all these requirements, and, in particular, aptamer-based sensors (also known as aptasensors) are listed as one of the most powerful type of biosensors [31]. In contrast to DNA biosensors, which cannot detect the allergen itself, aptasensors can directly indicate the presence of the analyte [32,33,34].

Among all electrochemical aptasensors, those based on structure-switching aptamers represent an encouraging methodology due to the fast, sensitive, and cheap determination of different target analytes [35]. This approach benefits from the variations in the conformation of the recognition aptamer, immobilized on the electrode surface [36,37]. This aptamer is usually altered at the 5’-end by a molecule containing a group able to link to the surface of the electrode (for example, a thiol) and labelled at the opposite side (3’-end) with a redox probe, for instance, methylene blue (MB) [38], which has been widely used as label for DNA-based biosensors [39]. This type of aptasensor, also known as a “folding-based aptasensor”, requires an alteration on the aptamer’s conformation when the analyte is present [40], allowing to detect changes in the efficiency of the electron transfer, which relies upon the spacing that exists between the surface of the electrode and the redox probe.

Conducting polymers, also known as the “fourth generation of polymeric materials”, have become competitive materials for biosensing applications [41,42]. The first time that a polymer was electrochemically prepared and characterized was in 1862 by Letheby [43], who carried out the electrolytic oxidation of a sulphatic solution of aniline, obtaining polyaniline. Since then, conducting polymers have been broadly used to modify electrodes for the development of chemical sensors and biosensors, because they conduct electricity very well, have an effective superficial area and are easy to prepare [44]. Considerably different conducting polymers have been stated [45], and polyaniline (PANI) [46], polyacetylene [47], polypyrrole (PPy) [48], and poly(3,4-ethylenedioxythiophene) (PEDOT) [49] are among the most widely used. Apart from them, another polymer that has attracted attention is poly-L-lysine (PLL), due to its versatility, good biocompatibility, stability, and good solubility in water [50,51]. It can be quickly prepared by the electropolymerization of L-lysine, an essential amino acid usually employed in protein biosynthesis [52]. It has been firstly described in 1977 by Shima and Sakai as a product of the fermentation of *Streptomyces* [53], and, after that, it has been widely used as food preservative. It also presents a great potential for using it as modifier of electrodes in electrochemical biosensors [54].

In this work, we present a folding-based aptasensor able to quantify β-lactoglobulin (β-LG) using graphite screen-printed electrodes (GSPEs). On their surface, a film made of a conducting polymer, poly-L-lysine, combined with electrogenerated gold nanoparticles (AuNPs) is used in order to improve the electrodic area. A thiolated aptamer that specifically recognizes β-LG is modified with methylene blue on the 3’-end, a redox probe, and is linked to the AuNPs via a thiol modification on the 5’-end. The strategy presented here takes advantage of: (i) the higher electroactive area of the GSPE when it is modified with PLL and AuNPs, (ii) the strong binding between AuNPs and the thiolated aptamer, (iii) the conformational changes the aptamer suffers when β-LG is present, and (iv) the differences in the electron transfer, which are dependent on the space existent in between MB and the modified electrode. This approach represents a sensitive, simple, and accurate methodology for the fast quantification of β-LG in real alimentary samples, using miniaturized and low-cost instrumentation.

## 2. Materials and Methods

### 2.1. Chemicals and Reagents

Di-sodium hydrogen phosphate (Na_2_HPO_4_), sodium dihydrogenphosphate dihydrate (NaH_2_PO_4_·2H_2_O), sodium chloride (NaCl), L-lysine hydrochloride (C_6_H_14_N_2_O_2_·HCl), tetrachloroauric acid (HAuCl_4_), sulfuric acid (H_2_SO_4_), and potassium chloride (KCl) were acquired from Merck (Milan, Italy). Potassium hexacyanoferrate (II) trihydrate (K_4_[Fe(CN)_6_]·3H_2_O), potassium hexacyanoferrate (III) (K_3_[Fe(CN)_6_]), Trizma^®^ Base (Tris), magnesium chloride hexahydrate (MgCl_2_·6H_2_O), β-lactoglobulin B (β-LG), and 6-mercapto-1-hexanol (MCH) were obtained from Sigma-Aldrich (Milan, Italy).

The thiolated aptamer used, modified in the 3’-end with methylene blue (MB), had the following sequence: 5’-HS-CGA CGA TCG GAC CGC AGT ACC CAC CCA CCA GCC CCA ACA TCA TGC CCA TCC GTG TGT G-MB-3’, and was obtained from Biomers.net (Ulm, Germany).

All the reagents used were of analytical grade and used as they were received, without the need of further purification. Milli-Q water (18 MΩ) was used to prepare all the solutions.

### 2.2. Apparatus

An AUTOLAB PGSTAT 10 potentiostat/galvanostat from Eco Chemie (Utrecht, The Netherlands) was used to carry out the electrochemical measurements, interfaced to a computer system and controlled by General Purpose Electrochemical System (GPES) software (Eco Chemie, Utrecht, The Netherlands).

The graphite screen-printed electrodes (GSPEs) used for the development of this aptasensor, which contained a silver pseudoreference electrode and a graphite auxiliary electrode, were purchased from EcoBioServices (Florence, Italy).

The microcentrifuge used for the real samples treatment was an Eppendorf^®^ miniSpin^®^, from Eppendorf (Hamburg, Germany).

### 2.3. Sensor Development

#### 2.3.1. Gold-Nanoparticles @ Poly-L-Lysine Electrodeposition

The surface of the working electrodes was, initially, modified by electropolymerization of L-lysine in order to create a poly-L-lysine (PLL) film following a procedure described by Kuralay and collaborators with some modifications [55]. Then, another modification of the polymer-modified electrodic surface was performed by the electrodeposition of gold nanoparticles (AuNPs). Both modifications have been performed using cyclic voltammetry (CV). Briefly, 50 µL of a 10 mM L-lysine solution in 50 mM phosphate buffer, 0.1 M NaCl, pH = 7.5 were dropped onto the electrochemical cell. Cyclic voltammograms were registered from –0.5 V to +1.5 V for 15 scans at a scan rate of 100 mV·s^−1^. The poly-L-lysine-modified GSPEs (PLL/GSPEs) were washed with 50 µL of 0.5 M H_2_SO_4_.

Then, AuNPs were electrogenerated by putting a drop of 50 µL of 0.5 mM HAuCl_4_ solution in 0.5 M H_2_SO_4_ on the PLL/GSPEs, and the potential was switched from −0.2 V to +1.2 V at 100 mV·s^−1^ for 15 cycles. Finally, the gold nanoparticles/poly-L-lysine-modified GSPEs (AuNPs@PLL/GSPEs) were washed three times with 100 µL of Milli-Q water, to remove the excess of free ions from the surface. It has been assessed that the modified GSPEs are stable at 4 °C in dry conditions for several weeks to use them in following experiments without affecting the analytical characteristics.

#### 2.3.2. Electrochemical Characterization of the Modified GSPEs

The modification of the GSPEs was monitored by cyclic voltammetry, dropping 50 µL of the redox probe [Fe(CN)_6_]^4−/3−^ (equimolar solutions of 5.0 mM in 0.1 M KCl). The potential was cycled from –0.4 V to +0.8 V using different scan rates (25, 50, 75, 100, 125, and 150 mV·s^−1^). The intensity of the current (i_p_, A) was measured and plotted against the square root of the scan rate (ν, V·s^−1^), showing a linear behaviour. The equation was fitted using the Randles–Sevcik equation [56,57], as in every reversible and diffusion-controlled process:

(1) $$i_{p}={0.446\;\mathrm{nFAC}}_{0}\sqrt{\frac{{\mathrm{nF}\nu D}_{0}}{\mathrm{RT}}}$$

where n is the number of electrons exchanged, A is the electrode surface area (cm^2^), C_0_ is the bulk concentration of the electroactive species (mol·cm^−3^), and D_0_ is the diffusion coefficient of the electroactive species (cm^2^·s^−1^) [58].

The sensors were considered as single use, so after each measurement, the electrodes were discarded.

#### 2.3.3. Aptamer Immobilization

The aptamer immobilization was carried out by depositing 1 µM of the thiolated aptamer in 50 mM Tris buffer solution (150 mM NaCl, 2 mM MgCl_2_, pH = 7.4) and letting it to react overnight. The aptamer was immobilized by chemisorption between AuNPs and the thiolated aptamer. After that, a self-assembled monolayer was formed with 1 mM 6-mercapto-1-hexanol solution in Milli-Q water and incubating it for 60 min, as previously described by our group [59]. Then, the aptasensors were washed with 0.1 M phosphate buffered saline (PBS) buffer solution, containing 150 mM NaCl (pH = 7.4).

#### 2.3.4. β-Lactoglobulin Detection

With the aim of obtaining the calibration curve for β-lactoglobulin, various protein concentrations have been tested. Several β-lactoglobulin solutions of increasing concentrations (between 0.1 ng·mL^−1^ and 10 ng·mL^−1^) in 0.1 M PBS buffer solution (150 mM NaCl, pH = 7.4) have been analyzed.

#### 2.3.5. Differential Pulse Voltammetry Measurements

Differential pulse voltammetry (DPV) measurements were carried out using 0.1 M PBS buffer solution, containing 150 mM NaCl (pH = 7.4) under the following conditions: modulation time: 0.02 s; interval time: 0.5 s; initial potential −0.60 V; end potential +0.15 V; step potential 0.005 V; modulation amplitude 0.10 V. The height of the resulting peak at around −0.03 V, corresponding to the oxidation of the MB attached to the aptamer, was taken as the analytical signal.

#### 2.3.6. Real Samples Analysis

Real samples were treated following a procedure described elsewhere [60]. Spike and recovery experiment was done in order to see if a real sample matrix affects the quantification of β-lactoglobulin, comparing it to the electrolyte solution used in the standard calibration curve (PBS). Biscuits and soya yoghourt were purchased in local markets.

The procedure was carried out as follows: 1 g of biscuit and 1 g of soya yoghourt were dissolved in 20 mL of 20 mM Tris-HCl (pH 8.0, containing 2% Tween-20) and stirred at room temperature for 5 hours. After that, the mixtures were centrifuged at 10,000 rpm for 15 min and the supernatant was collected. The samples were enriched with 1 and 5 ng·mL^−1^ of β-LG and the concentration was determined using the aptasensor. Finally, the recovery (%) of the concentration was calculated in the real samples.

## 3. Results and Discussion

### 3.1. Modification of GSPEs

**Figure 1:**
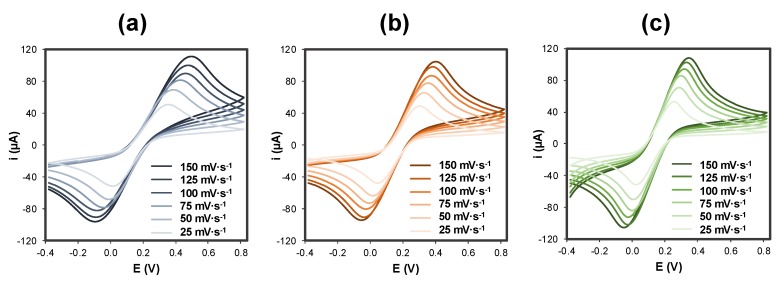
Cyclic voltammograms recorded with bare and modified GSPEs in an equimolecular solution of 5 mM ferro/ferricyanide ([Fe(CN)_6_]^4−/3−^) in 0.1 M KCl. (**a**) GSPE; (**b**) PLL/GSPE and (**c**) AuNPs@PLL/GSPE. Experimental parameters: potential range from −0.4 V to +0.8 V; scan rate: 25, 50, 75, 100, 125 and 150 mV·s^−1^.

The modification was evaluated in all the steps of the modification using cyclic voltammetry at different scan rates (25, 50, 75, 100, 125, and 150 mV·s^−1^) using the redox probe ferro/ferricyanide ([Fe(CN)_6_]^4−/3−^). Cyclic voltammograms obtained for bare GSPEs, poly-L-lysine-modified GSPEs (PLL/GSPE), and gold nanoparticles/poly-L-lysine-modified GSPEs (AuNPs@PLL/GSPE) in [Fe(CN)_6_]^4−/3−^ solutions are represented in Figure 1. The redox peaks recorded with PLL@AuNPs/GSPEs and PLL/GSPEs were higher than those obtained with bare GSPEs, as well as the reversibility.

This demonstrates the formation of polymer on the graphite surface. The electroactive surface area, which can be seen in Table 1, was calculated by applying the Randles–Sevcik equation using the slope of the linear regression obtained by plotting the current peak height against the square root of the scan rate.

The scan rate study depicted in Figure 1 shows that anodic and cathodic intensities (i_pa_ and i_pc_, respectively) increased with the scan rate, which was varied between 25 and 150 mV·s^−1^. Concerning the area of the electroactive surface, it can be seen in Table 1 that it increases in each step of the modification, being maximum when the GSPE is modified with both PLL and AuNPs.

### 3.2. Aptasensor Assay

**Figure 2:**
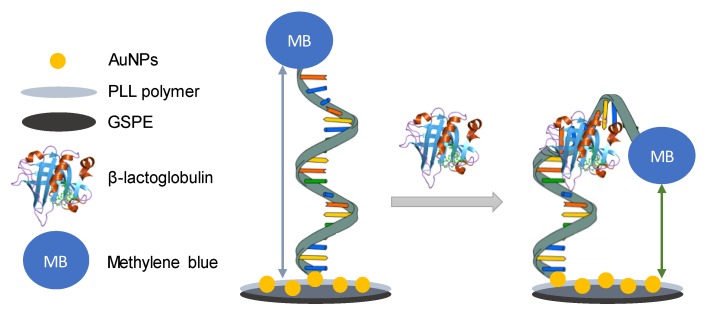
Schematic representation of the sensing strategy of the proposed aptasensor.

The aptasensor described here works with a folding-based mechanism (Figure 2), using a thiolated aptamer tagged with methylene blue (MB). The aptamer immobilization takes advantage of the covalent linking between thiolated groups and AuNPs. When there is no β-lactoglobulin, there is more distance between the redox probe (MB) and the electrode surface, so the signal is lower. However, when the analyte is added, the conformation of the aptamer changes, thus bringing the redox probe and the electrode surface together and increasing the signal, measured using differential pulse voltammetry (DPV).

For the purpose of obtaining the best analytical characteristics of this aptasensor, different parameters were optimized, as detailed in the following paragraphs.

#### 3.2.1. Electrolyte Solution

**Figure 3:**
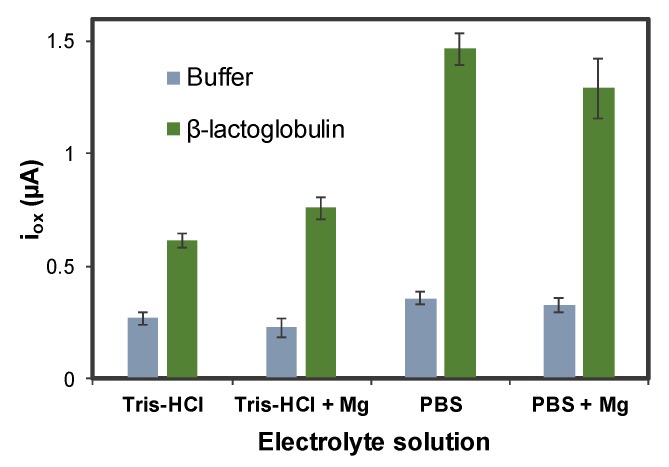
Optimization of the electrolyte solution. Experimental conditions: [β-LG] = 0 and 5 ng·mL^−1^, [Apt-MB] = 1 µM, incubation time: 45 min, oxidation DPV recorded between −0.60 V and +0.15 V, using the following conditions: modulation time: 0.02 s; interval time: 0.5 s; step potential 0.005 V; modulation amplitude 0.10 V. Solutions made in 0.1 M PBS, 150 mM NaCl pH = 7.4. Data are given as average ± SD (n = 3).

Different buffer solutions were tested for the MB detection with the objective of obtaining the highest signal-to-noise ratio: 50 mM Tris-HCl, 150 mM NaCl pH = 7.5 (with and without 2 mM MgCl_2_) and 0.1 M PBS, 150 mM NaCl pH = 7.4 (with and without 2 mM MgCl_2_). In Figure 3, the results of this optimization are depicted, testing a blank solution of buffer (background electrolyte) and 5 ng·mL^−1^ of β-LG in each case.

As shown in Figure 3, the highest signal-to background ratio was obtained when PBS-based buffers were used. In particular, the signal-to-noise ratio is bigger without MgCl_2_, so this buffer solution was selected for the following measurements. This suggests that magnesium ions can influence the conformation of the aptamer, and therefore its interaction with the protein, taking into account the decrease in the mobility of the aptamer and the water molecules when Mg^2+^ is present, as reported by Alexander D. MacKerell [61].

#### 3.2.2. Aptamer Concentration

**Figure 4:**
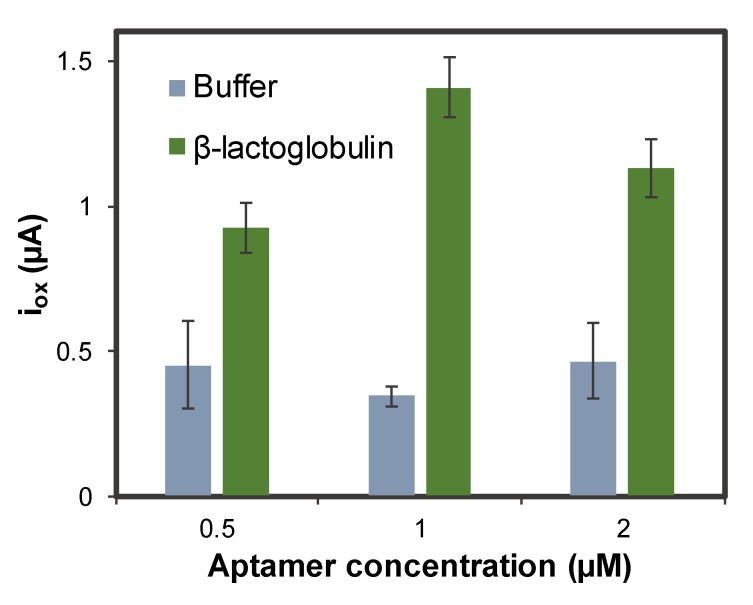
Optimization of the concentration of aptamer immobilized on the AuNPs@PLL/GSPE. Experimental conditions: [β-LG] = 0 and 5.0 ng·mL^−1^, incubation time: 45 min, oxidation DPV recorded between –0.60 V and +0.15 V, using the following conditions: modulation time: 0.02 s; interval time: 0.5 s; step potential 0.005 V; modulation amplitude 0.10 V. Solutions made in 0.1 M PBS, 150 mM NaCl pH = 7.4. Data are given as average ± SD (n = 3).

A crucial stage in the development of the aptasensor is the concentration of aptamer immobilized. Different concentrations of anti-β-LG aptamer labeled with MB (0.5, 1, and 2 µM) were immobilized on the AuNPs@PLL/GSPE, and the aptasensor response is shown in Figure 4.

The best concentration was chosen to be 1 µM because of the higher signal-to-background ratio obtained. The precision was also better in this case.

#### 3.2.3. Incubation Time

**Figure 5:**
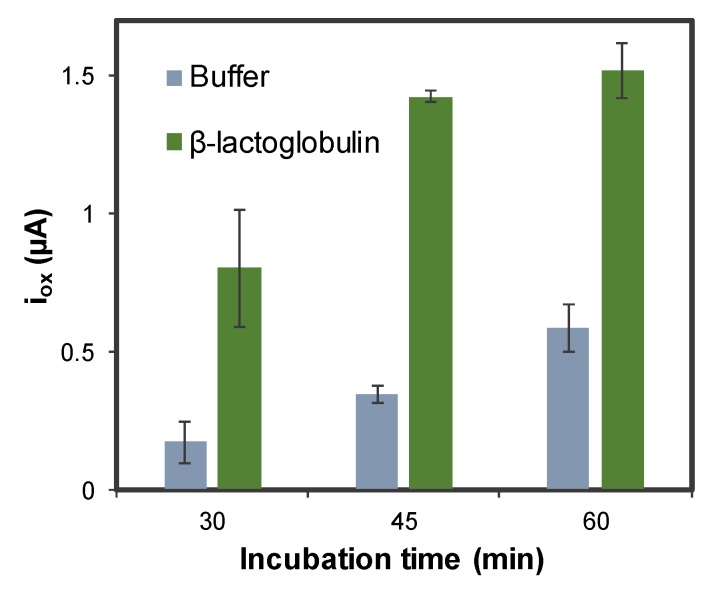
Optimization of the incubation time (aptamer-target interaction) in the AuNPs@PLL-GSPE. Experimental conditions: [β-LG] = 0 and 5.0 ng·mL^−1^, [Apt-MB] = 1 µM, oxidation DPV recorded between −0.60 V and +0.15 V, using the following conditions: modulation time: 0.02 s; interval time: 0.5 s; step potential 0.005 V; modulation amplitude 0.10 V. Solutions made in 0.1 M PBS, 150 mM NaCl pH = 7.4. Data are given as average ± SD (n = 3).

Incubation time is a very important parameter that can affect the measurements, inasmuch as it can affect to an appropriate conformational change in the aptamer structure when it binds the analyte. The results for incubation times of 30, 45, and 60 min are shown in Figure 5. As can be seen, a bigger signal-to noise ratio is obtained for 45 min, that being the time chosen as the optimum incubation time.

### 3.3. β-Lactoglobulin Detection in Standard Solutions

**Figure 6:**
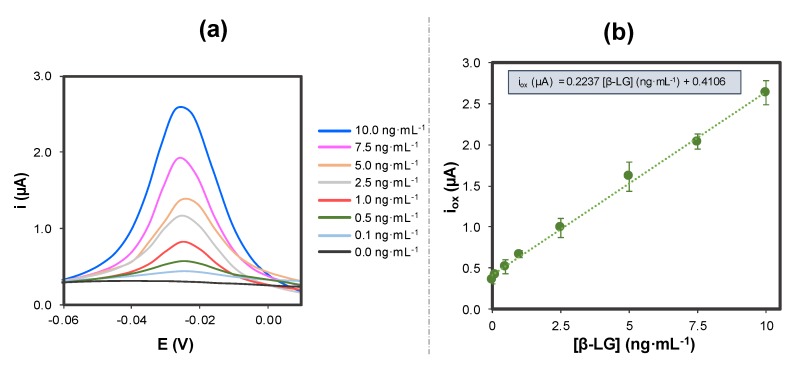
(**a**) DPV measurements for increasing concentrations of β-LG under the optimized conditions; (**b**) calibration curve for β-LG on AuNPs@PLL/GSPEs. Experimental conditions: [Apt-MB] = 1 µM, Incubation time: 45 min, oxidation DPV recorded between –0.60 V and +0.15 V, using the following conditions: modulation time: 0.02 s; interval time: 0.5 s; step potential 0.005 V; modulation amplitude 0.10 V. Solutions made in 0.1 M PBS, 150 mM NaCl pH = 7.4. Data are given as average ± SD (n = 3).

The influence of the concentration of β-LG on the analytical signal (taking into account the voltammetric peak at around –0.03 V) under the optimized conditions was evaluated, as shown in Figure 6. Different β-LG concentrations ranging from 0.10 to 10.0 ng·mL^−1^ were analyzed.

The current peak intensity resulted to be directly proportional to β-LG concentration, so the experimental data were fitted with a linear relationship in the range studied, in accordance with the following equation:

i_ox_ (µA) = 0.2237 (µA·mL·ng^−1^)·[β-LG] (ng·mL^−1^) + 0.4106 (µA) R^2^ = 0.997 (2)

A limit of detection (LOD), calculated as three times the standard deviation of the intercept divided by the slope, was found to be 0.09 ng·mL^−1^. This LOD is below the threshold established for proteins in cow’s milk [8,62] so it would be very suitable for the determination of trace concentrations in food in order to avoid undesired reactions in allergenic patients. In addition, the levels of β-LG assayed in this work are close (and even beneath) to those achieved using alternative approaches based on liquid chromatography combined with mass spectrometry [22], ELISA [60], capillary-electrophoresis, and laser-induced fluorescence [63] or magneto-immunoassays [64]. 

Apart from that, the reproducibility of the method was very good, with a relative standard deviation (RSD) below 13% (obtained for three repetitive measurements for all the concentrations tested).

Before using the biosensor for analyzing real samples, the effect of different interferences that can affect the signal of this aptasensor was studied. One of the main components present in milk samples that can interfere is casein. In this way, individual solutions of β-LG and casein and a mixture of both were used.

**Figure 7:**
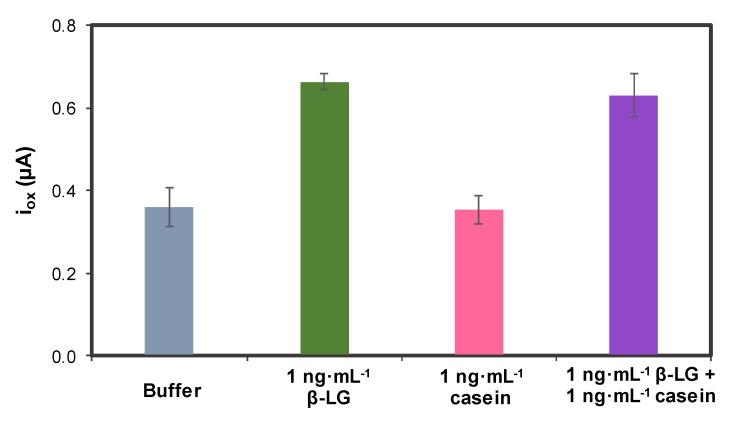
Study of the possible interference caused by casein. Experimental conditions: [Apt-MB] = 1 µM, Incubation time: 45 min, oxidation DPV recorded between –0.60 V and +0.15 V, using the following conditions: modulation time: 0.02 s; interval time: 0.5 s; step potential 0.005 V; modulation amplitude 0.10 V. Solutions made in 0.1 M PBS, 150 mM NaCl pH = 7.4. Data are given as average ± SD (n = 3).

As can be seen in Figure 7, casein on its own did not produce any interference, giving a signal very similar to the one obtained with buffer. In the case of the mixture, the presence of casein hardly affects the β-LG signal, demonstrating the high specificity of the aptamer employed.

### 3.4. β-Lactoglobulin Detection in Real Samples

The main goal for demonstrating the applicability of the aptasensor is analysis in real food samples.

The performance of the aptasensor towards the analysis of real food samples has been verified using commercially available biscuits and soya yoghourt, and its response has been tested using the same conditions used for the β-LG calibration curve. 

The samples were treated as specified in the Experimental Section, and a spike and recovery experiment was performed. The results are summarized in Table 2. The recovery rates of the concentration reveal that the matrix doesn’t affect the methodology to a great extent. This opens up the way to β-LG quantification in food samples with high accuracy for the detection of allergens.

## 4. Conclusions

In this work, a simple and sensitive aptasensor for the detection of β-lactoglobulin, one of the most important proteins found in milk, has been designed. The aptasensor developed here is built on disposable graphite screen-printed electrodes modified with a conducting polymer (poly-L-lysine) and gold nanoparticles. The modification of the biosensor surface was carried out by the electropolymerization of L-lysine, in a first step, and the electrogeneration of AuNPs using gold tetrachloroauric acid.

The thiolated aptamer used in this aptasensor is modified with the redox probe methylene blue at the 3’-end. The strategy presented in this work benefits from the conformational changes of the aptamer when β-LG is present, and as a result, from changes in the electron transfer, hanging on the distance between methylene blue and the electrodic surface. A good linear relationship between the peak current values and β-LG concentration in the range between 0.10 and 10 ng·mL^−1^, with a limit of detection of 0.09 ng·mL^−1^, was obtained. The aptasensor was also applied for the determination of spiked β-LG in real food samples found in local supermarkets.

## Figures and Tables

**Table 1:** Electroactive surface area at the different steps of the modification of the GSPEs, calculated from the CV scans performed in 5 mM [Fe(CN)6]^4−/3−^ equimolecular redox probe in 0.1 M KCl.

	**A_anodic_ (mm^2^)**	**A_cathodic_ (mm^2^)**	**A_average_ (mm^2^)**	**RSD (%)**
GSPE^1^	3.57	3.82	3.69	4.8
PLL/GSPE^2^	4.89	4.42	4.65	7.2
AuNPs@PLL/GSPE^3^	5.93	5.30	5.61	7.8

^1^ GSPE: Graphite screen-printed electrodes. ^2^ PLL/GSPE: Poly-L-lysine modified GSPE. ^3^ AuNPs@PLL/GSPE: Gold nanoparticles and PLL-modified GSPE.

**Table 2:** Spike and recovery experiment data. Concentrations of 1.0 and 5.0 ng·mL^−1^ of β-LG were spiked in biscuit and soya yoghourt samples (n = 3 for each). The recovery (%) was calculated comparing the concentration obtained with the samples with that obtained in PBS buffer, using the optimized conditions.

Samples	Spiked [β-LG] (ng·mL^−1^)	Found [β-LG] (ng·mL^−1^)	Recovery (%)
Biscuit	1.0	1.17	117
	5.0	5.18	103
Soya yoghourt	1.0	1.16	116
	5.0	4.78	95
